# The neural correlates of perceived social support and its relationship to psychological well-being

**DOI:** 10.3389/fnbeh.2023.1295668

**Published:** 2024-01-08

**Authors:** Huanhua Lu, Yiying Song, Xu Wang, Jia Liu

**Affiliations:** ^1^School of Marxism, China University of Geosciences, Beijing, China; ^2^Beijing Key Laboratory of Applied Experimental Psychology, Faculty of Psychology, National Demonstration Center for Experimental Psychology Education, Beijing Normal University, Beijing, China; ^3^School of Life Sciences, Beijing University of Chinese Medicine, Beijing, China; ^4^Tsinghua Laboratory of Brain and Intelligence, Department of Psychology, Tsinghua University, Beijing, China

**Keywords:** perceived social support, middle temporal gyrus, voxel-based morphometry, psychological well-being, individual difference

## Abstract

**Introduction:**

Perceived social support is considered to play a significant role in promoting individuals’ health and well-being, and yet the neural correlates of perceived social support were not fully understood. An exploration of the neural correlates of individual differences in the SPS can help us to gain more comprehensive understanding about the neural correlates of perceived social support. What’s more, our study will explore the relationship among perceived social support, brain regions, and psychological well-being, which may provide new insights into the neural correlates underlying the relationship between perceived social support and psychological well-being from the perspective of cognitive neuroscience.

**Methods:**

Herein, we used the Social Provisions Scale to assess individuals’ perceived social support, and magnetic resonance imaging was used to measure the gray matter (GM) volume of the whole brain. What’s more, we also measured psychological well-being using the Psychological Well-Being Scale, and mediation analysis was used to explore the relationship among perceived social support, brain regions, and psychological well-being.

**Results:**

The voxel-based morphometry analysis of the whole brain revealed that perceived social support was positively correlated with GM volume of the left middle temporal gyrus (MTG). The finding indicated that a person with greater GM volume in the left MTG perceived more social support. More importantly, the left MTG GM volume observed above was also associated with psychological well-being, and the link between the two was mediated by perceived social support.

**Discussion:**

These results revealed the importance of MTG for perceived social support and psychological well-being, and also suggested that perceived social support might explain the relationship between MTG and psychological well-being.

## Introduction

Humans are social creatures. Social support has been widely shown to be reliably associated with our happiness and health (Taylor, 2011). Social support can be divided into actual receiving social support and subjective perceived social support in nature (Wethington and Kessler, 1986; Haber et al., 2007; Uchino, 2009). Perceived social support is the support that the individual believes to be available, whether or not it is in fact available (Wethington and Kessler, 1986). Perceived social support has been proven to be more strongly related to health and well-being than actual receiving social support (Wethington and Kessler, 1986; Rees and Freeman, 2007; Uchino, 2009; Melrose et al., 2015).

Researchers have developed a number of reliable and standardized scales to assess perceived social support (Cutrona and Russell, 1987; Zimet et al., 1990; Sherbourne and Stewart, 1991; Ryff and Singer, 1996). These scales differed in their theoretical foundations, focus, and the dimensions of social support they measured. For instance, the Multidimensional Scale of Perceived Social Support (MSPSS) was based on a general conceptualization of social support and was designed to measure three sources of support: family, friends, and significant others (Zimet et al., 1990). The Medical Outcomes Study Social Support Survey (MOS-SSS) was grounded in the stress-buffering model and measured four different types of functional support (tangible, emotional/informational, positive social interaction, and affectionate) (Sherbourne and Stewart, 1991). In contrast, the Social Provisions Scale (SPS) was based on the social provisions model which posited that social relationships served specific functions or provisions that were essential for coping with stress, adapting to challenges, and achieving a sense of belonging and security, and then contributed to an individual’s overall well-being (Weiss, 1974). The SPS was designed to assess the extent to which individuals perceive that their social relationships fulfill six types of social provisions: social integration, reassurance of worth, attachment, reliable alliance, guidance, and opportunity for nurturance (Cutrona and Russell, 1987). In short, unlike the MSPSS that measured sources of social support (Zimet et al., 1990), the SPS assessed the social provisions individuals sought in their relationships (Cutrona and Russell, 1987); while compared to the MOS-SSS (Sherbourne and Stewart, 1991), the SPS measured a wider types of social support (Cutrona and Russell, 1987). Therefore, the SPS reveals structures and functions of perceived social support distinct from other scales.

Previous research has demonstrated that perceived social support, whether measured by the MSPSS or the MOS-SSS or the SPS, was closely related to social network size (Cutrona, 1986; Russell et al., 1997; Reinhardt and Blieszner, 2000; Chan and Lee, 2006; Lu and Hampton, 2017; Bi et al., 2022), which refers to the total number of individuals with whom a person maintains some form of social connection or interaction. And social network size was found to be associated with many brain regions including the medial prefrontal cortex (MPFC), anterior cingulate cortex (ACC), orbitofrontal cortex (OFC), posterior cingulate cortex (PCC), insula, amygdala, hippocampus, middle temporal gyrus (MTG), precuneus and superior temporal sulcus (STS) (Bickart et al., 2011; Kanai et al., 2011; Brink and Ghazanfar, 2012; Heide et al., 2014; Molesworth et al., 2015; Blumen and Verghese, 2019; Spreng et al., 2020; Peer et al., 2021). However, till now only some studies have examined the neural correlates of perceived social support (Che et al., 2014a,b; Sato et al., 2016; Cotton et al., 2020). One study in which elderly people were selected as participants and perceived social support was determined by the MOS-SSS has found that a gray matter (GM) network including prefrontal, hippocampal, amygdala, cingulate, and thalamic regions was involved in perceived social support (Cotton et al., 2020). Other studies using the MSPSS have revealed that perceived social support was associated with the GM volume of amygdala and posterior cingulate cortex (Che et al., 2014a; Sato et al., 2016), as well as synchronous activities among brain regions within the default mode network (DMN) (Che et al., 2014b).

The SPS has been widely used and shown to be associated with self-esteem, mental health, and well-being (Bell and Gonzalez, 1988; Cutrona, 1989; Chen et al., 2012; Kvaal et al., 2014; Scardera et al., 2020; Rippon et al., 2022; Lu et al., 2023; Xin, 2023). Researchers often consider the SPS as a valuable tool for understanding the nuanced ways in which social support contributes to individuals’ well-being and adaptation to life circumstances (Russell et al., 1984; Bell and Gonzalez, 1988; Cutrona, 1989; Mancini and Blieszner, 1992; Gottlieb and Bergen, 2010; Chen et al., 2012; Kvaal et al., 2014; Perera, 2015; Scardera et al., 2020; Rippon et al., 2022; Lu et al., 2023; Xin, 2023). However, as far as we know, there is no research that examined the neural correlates of perceived social support measured with the SPS. Therefore, an exploration of the neural correlates of individual differences in the SPS can help us to gain more comprehensive understanding about the neural correlates of perceived social support.

Psychological well-being is the perfect experience of maximizing the meaning of one’s life when one strives to realize one’s own potential and strengths (Ryff and Singer, 1996). Numerous previous investigations have revealed that perceived social support was related to psychological well-being (Huppert, 2010; Orehek and Lakey, 2011; Lakey, 2013; Kalpana, 2016; Szkody and Mckinney, 2019). Brain imaging studies have revealed that psychological well-being was related to multiple brain regions, involving the prefrontal cortex, cingulate gyrus, MTG, amygdala, insula, striatum, and hippocampus (Lewis et al., 2013; Kong et al., 2014; Luo et al., 2017; Costa et al., 2019; King, 2019), some of which have been reported to be associated with social network size and social support (Bickart et al., 2011; Heide et al., 2014; Che et al., 2014a; Molesworth et al., 2015; Sato et al., 2016; Blumen and Verghese, 2019; Cotton et al., 2020). Therefore, it is likely that there are some brain regions linking perceived social support with psychological well-being. Our study will explore the relationship among perceived social support, brain regions, and psychological well-being, which may provide new insights into the neural correlates underlying the relationship between perceived social support and psychological well-being from the perspective of cognitive neuroscience.

Here, we aimed to examine the neural correlates of perceived social support and to further explore its relationship with psychological well-being. First, we used the SPS to determine perceived social support, and then voxel-based morphometry (VBM) was used to examine the neural correlates of perceived social support. What’s more, we also measured psychological well-being using the Psychological Well-Being Scale (PWBS) (Ryff and Singer, 1996), and mediation analysis was used to explore the relationship among perceived social support, brain regions, and psychological well-being.

## Materials and methods

### Participants

Two hundred and forty-four college students (females 123, males 121; range of the age: 19–25; mean age = 22.65) volunteered for our study, which is part of an ongoing research project (i.e., the Gene–Environment Brain and Behavior study) (Kong et al., 2014; Song et al., 2015; Lu et al., 2018). All the participants were Han Chinese. Participants were asked to self-report “Have you ever been diagnosed with any mental illness or psychological disorders? None of the 244 participants reported mental illness or psychological disorders. All experimental protocols were granted approval by the IRB (Institutional Review Board) of Beijing Normal University (BNU). All participants signed an informed consent form before participation in the experiment.

### Assessment of perceived social support

This assessment was performed using the SPS (Cutrona and Russell, 1987). The SPS measures the degree of which a person perceives his or her social ties as giving social support. It has 24 questions and can be divided into 6 dimensions such as social integration (SI), reassurance of worth (ROW), attachment (AT), reliable alliance (RA), guidance (GU), and opportunity for nurturance (OFN) (Cutrona and Russell, 1987). The SPS is based on a six-point scale and has been shown to have a good convergent and discriminant validity (Oluwatomiwo, 2015; Perera, 2015). In this study, the internal consistency of the SPS (Cronbach’s α =0.927) and its sub-dimensions was acceptable (SI: 0.770; ROW:0.728; AT: 0.797; RA: 0.693; GU:0.759; OFN: 0.718).

### Assessment of psychological well-being

The PWBS (Ryff and Singer, 1996) was used to measure the psychological well-being of the participants. The scale contains 84 items and is divided into six dimensions: self-acceptance (SA); positive relationships with others (PRWO); purpose in life (PIL); environmental mastery (EM); autonomy (AU); and personal growth (PG) (Ryff and Singer, 1996). The questionnaire is scored on a 6-point scale. It is widely used and has been proved to have sound reliability and validity (Lindfors et al., 2006; Gallagher et al., 2010). The total score of the scale was used as the indicator of psychological well-being, with higher score representing greater well-being. In this study, the internal consistency of the PWBS (Cronbach’s α =0.954) and all its sub-dimensions was good (SA:0.890; PRWO:0.898; PIL: 0.850; EM:0.864; AU:0.833; PG:0.818).

### Assessment of subjective socioeconomic status

The MacArthur Scale of Subjective Social Status (Goodman et al., 2001) was used to measure the socioeconomic status (SES) of the participants. In the scale, a ten-rung “social ladder “was presented to the participants, in which the highest rung of the ladder represented the best conditions in terms of money, occupation and education, and the lowest rung represented the worst conditions (Goodman et al., 2001). Participants were asked to point out “which rung of the ladder best represents your family’s socioeconomic status.”

### MRI data acquisition

A Siemens 3 T Trio scanner (MAGENTOM Trio with a Tim system) equipped with a 12-channel cranial phased-array coil was used for data acquisition. A high-resolution 3D T1-weighted structural image scan was performed using an inversion preparation gradient echo sequence. The scanning time for each participant was 8 min. The scanning parameters were as follows: bandwidth = 190 Hz/pixel, the number of slices = 128, TR/TE/TI = 2.53 s/3.39 ms/1.1 s, flip angle = 7-degree, slice thickness = 1.33 mm.

### VBM analysis

We used VBM to examine the neural correlates of perceived social support, and the VBM-DARTEL method in the SPM8 toolbox was used for the operation. The raw DICOM data were first converted to SPM-recognizable NII format using the MRIcron package, and then the SPM8 package was enabled with the following processing steps: (i) manually adjusted the image position to locate the origin at the line of anterior–posterior association; (ii) used unified segmentation approach (Ashburner and Friston, 2005) to segment the image into GM, white matter and cerebrospinal fluid; (iii) applied the DARTEL algorithm (Ashburner, 2007) to generate the average template of all participants, and the average template was aligned to the MNI standard space; (iv) the GM volume images were smoothed with a full width and height (FWHM) 8 mm smoothing kernel to improve the signal-to-noise ratio of the images and make the data more normally distributed for subsequent statistical analysis.

### Statistical analyses

Firstly, a general linear model (GLM) was used to examine the relationship between perceived social support and GM volume of each voxel within the whole brain. The total score of the SPS was used as the independent variable, and the dependent variable was GM volume of each voxel within the whole brain, with sex and total GM volume as covariates. The regression model was run for every voxel, and in each model we tested whether the GM volume of each voxel was associated with perceived social support. And then statistical parametric maps were obtained from whole brain voxel-wise analyses with setting the voxel-wise threshold at *p* < 0.05. To control for the multiple comparisons inherent in the statistical analysis of tens of thousands of brain voxels, we carried out a cluster-size threshold adjustment with a Monte Carlo simulation which was widely used in neuroimaging literature for multiple comparisons correction (Goldin et al., 2005; Moses-Kolko et al., 2010; Boehme et al., 2014). The Monte Carlo simulation showed that a cluster size greater than 454 voxels with setting the voxel-wise threshold at *p* < 0.05 was able to protect against a cluster-level false-positive rate of 5%. Next, we extracted the GM volume of brain regions observed above. Then, we randomly divided the whole sample into two groups, one as exploratory sample and the other as confirmatory sample. The regression model with the left MTG GM volume as the dependent variable, the total score of the SPS as the independent variable, sex, age, SES and total GM volume as covariates was run in both groups to verify the reliability of the above findings.

Finally, we examined the relationship among perceived social support, the brain regions observed above, and psychological well-being. First, we tested the relationship between the two behavioral variables measured, and examined whether brain regions observed above were associated with psychological well-being, and then, a mediation analysis was performed to explore the relationship among above three. In the mediation analysis, bootstrap simulation (*n* = 5,000) was used to test the significance of indirect effect.

## Results

Table 1 presented the descriptive statistics of the SPS and the PWBS. Higher scores on the SPS represented more perceived social support; higher scores on the PWBS represented greater psychological well-being. The range of the SPS scores was 81 to 143, and the range of the PWBS scores was 273–432, the kurtosis and skewness of the two behavioral measures were between −1 and 1, indicating that the data for both behavioral measures were normally distributed. Sex differences in the scores of the two behavioral measures were tested, and not any significant sex difference in psychological well-being was found (*t* = 0.56, *p* = 0.577), while the perceived social support of female was significantly higher than that of male (*t* = 2.83, *p* = 0.005).

**Table 1:** Descriptive statistics of all behavioral measures.

Measures	*M*	SD	Range	Skewness	Kurtosis
SPS	112.18	12.59	81–143	0.028	−0.525
PWBS	346.12	34.58	273–432	0.066	−0.643

M, mean; SD, standard deviation.

First, we examined the neural correlates of perceived social support. We carried out multiple regression analysis for every voxel within the whole brain. The findings showed that only the GM volume in the left MTG (MNI coordinates: 79, 53, 27; cluster size: 625; Figure 1) was significantly positively associated with perceived social support after whole brain correction. We extracted the GM volume of the left MTG observed above, and regression analysis showed that the left MTG GM volume could significantly predict perceived social support (*β* = 0.273, *p* = 0.002), with sex, age, SES and the total GM volume as covariates. To verify the reliability of the above findings, we randomly divided the sample into two groups, one as exploratory sample and the other as confirmatory sample. In the exploratory sample (*N* = 126), regression analysis showed that the left MTG GM volume could significantly predict perceived social support (*β* = 0.305, *p* = 0.019), with sex, age, SES and the total GM volume as covariates. In the confirmatory sample (*N* = 118), the similar result was found (*β* = 0.286, *p* = 0.024). Hence, it indicated that the above findings were reliable.

**Figure 1:**
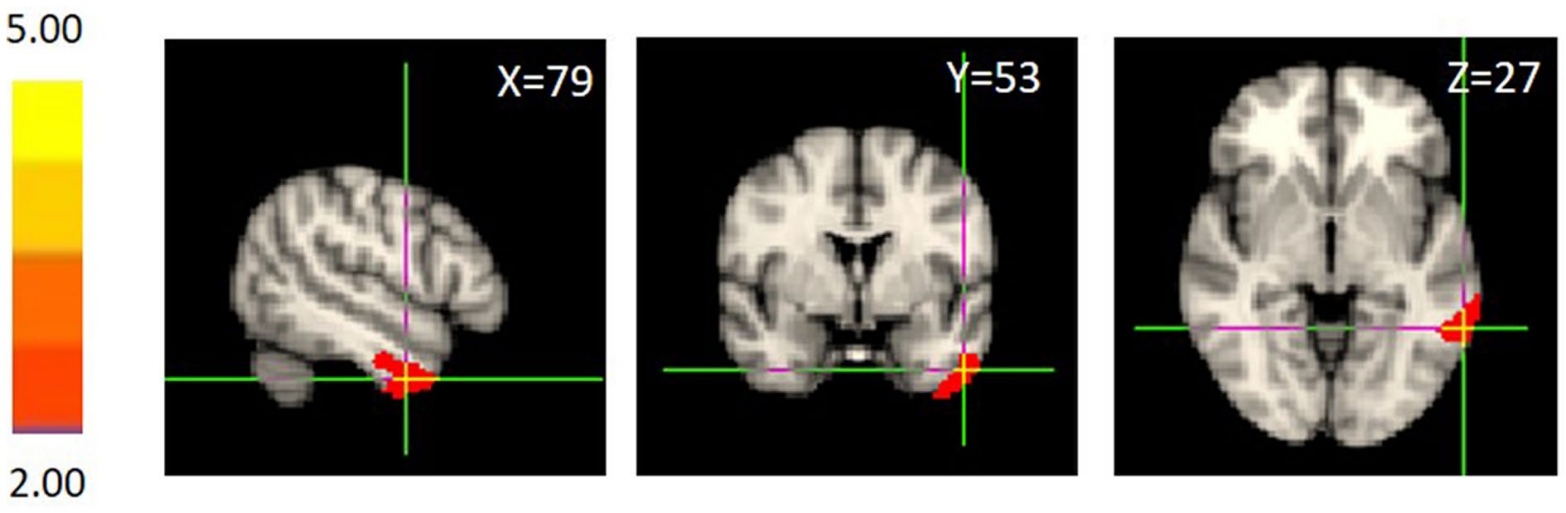
Brain regions correlated with perceived social support assessed by the SPS. When controlling for the total GM volume and sex, the left MTG GM volume was significantly positively associated with perceived social support after whole brain correction.

**Figure 2:**
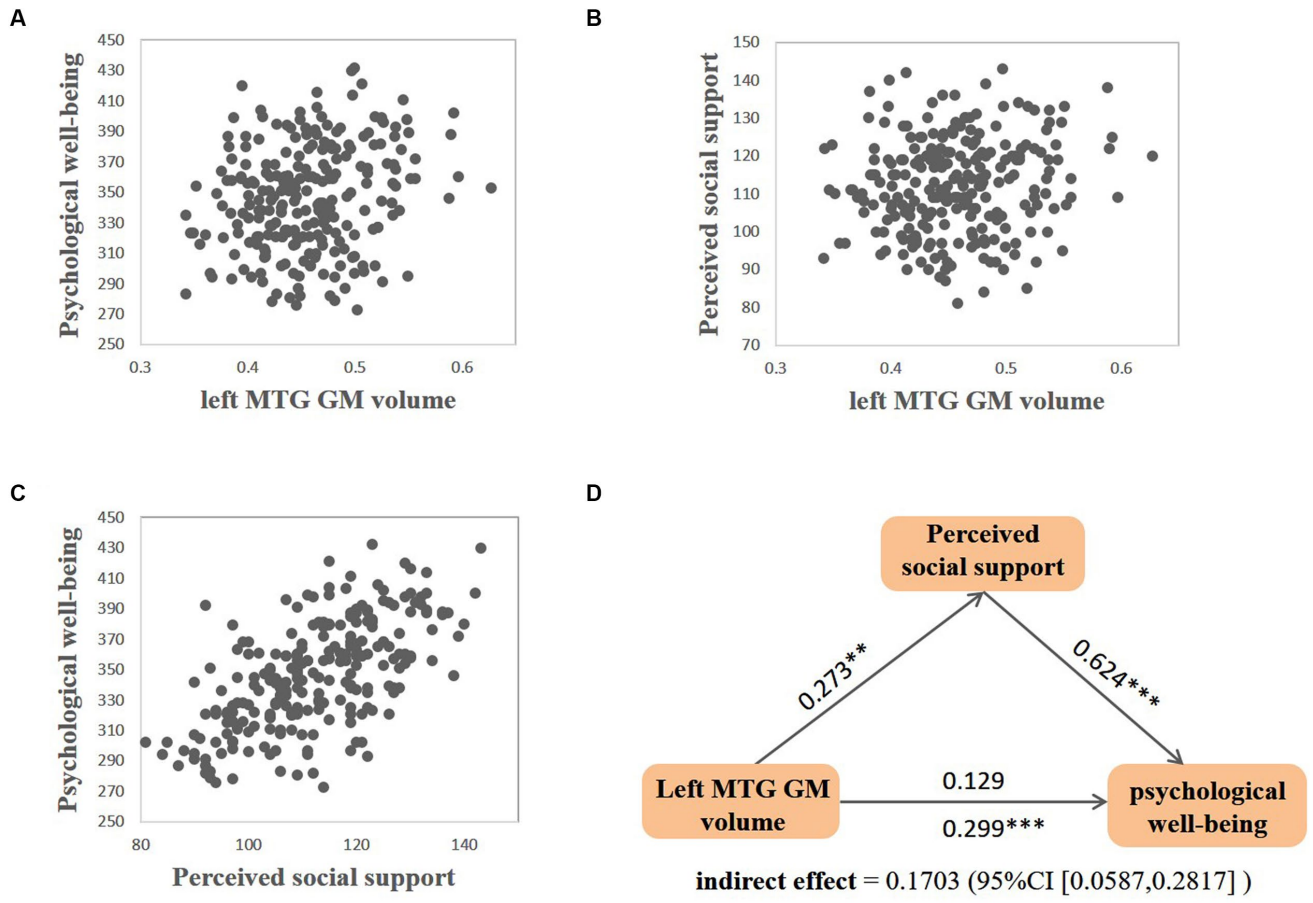
Relationship between the left MTG GM volume, perceived social support, and psychological well-being. Scatter plots for correlation between **(A)** the left MTG GM volume and psychological well-being, **(B)** the left MTG GM volume and perceived social support, **(C)** perceived social support and psychological well-being. **(D)** perceived social support mediated the relationship between the left MTG GM volume and psychological well-being. This is the path diagram for the mediation analysis with sex, age, SES and the total GM volume as covariates. All path coefficients are standardized betas. For the association between the left MTG GM volume and psychological well-being, coefficient below the arrow represents the original correlation, coefficient above the arrow represents the correlation after adding mediating variables. **p < 0.01; ***p < 0.001.

Further, we explored the relationship among perceived social support, the left MTG GM volume, and psychological well-being. First, regression analysis showed that perceived social support was significantly positively associated with psychological well-being after controlling for sex, age and SES (β = 0.644, *p* < 0.001). Next, we tested whether the GM volume of the left MTG observed above was significantly associated with psychological well-being. The regression analysis showed that the left MTG GM volume could significantly predict psychological well-being (*β* = 0.299, *p* < 0.001), with sex, age, SES and the total GM volume as covariates. Finally, a mediation analysis with the left MTG GM volume as the independent variable, psychological well-being as the dependent variable, perceived social support as a mediating variable, sex, age, SES and the total GM volume as covariates was conducted. The findings revealed that the total effect between the left MTG GM volume and psychological well-being was significant (*β* = 0.299, *p* < 0.001, Figure 2), but when perceived social support was added as a mediator, the association between the left MTG GM volume and psychological well-being was not significant (*β* = 0.129, *p* = 0.073, Figure 2). Bootstrap simulation (*n* = 5,000) verified that the indirect effect of the left MTG GM volume on psychological well-being through perceived social support was significant (95% CI = [0.0587, 0.2817], Figure 2).

The other mediation analysis was also performed, with the perceived social support as the independent variable, psychological well-being as the dependent variable, the left MTG GM volume as a mediator, sex, age, SES and the total GM volume as covariates. The finding revealed that the left MTG GM volume did not significantly mediate the relationship between perceived social support and psychological well-being (95% CI = [−0.0021, 0.0443]). The results further confirmed the mediating role of perceived social support for the relationship between the left MTG GM volume and psychological well-being.

Because the PWBS contained items related to social relationship, we made a supplementary data analysis in which we removed 14 items related to social relationship from the PWBS (e.g., “I know that I can trust my friends, and they know they can trust me”; “I feel like I get a lot out of my friendships”) and the total score of the remaining 70 items was used as a new indicator of psychological well-being. Regression analysis showed that perceived social support was still significantly associated with the new indicator of psychological well-being after controlling for sex, age and SES (*β* = 0.561, *p* < 0.001); and the left MTG GM volume could still significantly predict the new indicator of psychological well-being (*β* = 0.308, *p* = 0.001), with sex, age, SES and the total GM volume as covariates. A mediation analysis showed that perceived social support could still significantly mediate the relationship between the left MTG GM volume and the new indicator of psychological well-being (indirect effect = 0.1463, 95% CI = [0.0523, 0.2431]), with sex, age, SES and the total GM volume as covariates.

Moreover, we used correlation analyses to examine the relationship between the sub-dimensions of perceived social support and psychological well-being, as well as the relationship between perceived social support and the sub-dimensions of psychological well-being. The results showed that there was a positive correlation between each of the sub-dimensions of perceived social support and psychological well-being (SI: *r* = 0.523, *p* < 0.001; ROW: *r* = 0.613, *p* < 0.001; AT: *r* = 0.504, *p* < 0.001; RA: *r* = 0.414, *p* < 0.001; GU: *r* = 0.450, *p* < 0.001; OFN: *r* = 0.532, *p* < 0.001). Also, there was a significant positive correlation between perceived social support and five sub-dimensions of psychological well-being (SA: *r* = 0.521, *p* < 0.001; PRWO: *r* = 0.741, *p* < 0.001; PIL: *r* = 0.518, *p* < 0.001; EM: *r* = 0.514, *p* < 0.001; PG: *r* = 0.397, *p* < 0.001), but the correlation with AU was not significant (*r* = 0.093, *p* = 0.148). Further, regression analysis showed that the left MTG GM volume significantly predicted the five sub-dimensions of psychological well-being (SA: β = 0.246, *p* = 0.007; PRWO: β = 0.174, *p* = 0.052; PIL: β = 0.263, *p* = 0.004; EM: β = 0.189, *p* = 0.039; PG: β = 0.238, *p* = 0.009), with sex, age, SES and the total GM volume as covariates. Finally, we performed five mediation models with the left MTG GM volume as the independent variable, perceived social support as the mediator variable, the five sub-dimensions of psychological well-being (SA, PRWO, PIL, EM, and PG) as the dependent variable, respectively, and sex, age, SES and the total GM volume as covariates. The results showed that the indirect effects of all five mediation models were significant (Table 2), that is, perceived social support mediated the relationship between the left MTG GM volume and each of the five sub-dimensions of psychological well-being (SA, PRWO, PIL, EM, and PG).

**Table 2:** PSS mediated the relationship between the left MTG GM volume and each of the five sub-dimensions of psychological well-being.

Path	Indirect effect	Bootstrap SE	95% Confidence interval	
			LLCI	ULCI
Left MTG-PSS-EM	0.1397	0.0461	0.0537	0.2337
Left MTG-PSS-PG	0.1059	0.0390	0.0367	0.1891
Left MTG-PSS-PRWO	0.2010	0.0646	0.0769	0.3303
Left MTG-PSS-PIL	0.1388	0.0478	0.0471	0.2366
Left MTG-PSS-SA	0.1417	0.0477	0.0502	0.2399

MTG, middle temporal gyrus; PSS, perceived social support; EM, Environmental Mastery; PG, Personal Growth; PRWO, Positive Relationships with Others; PIL, Purpose in Life; SA, Self-Acceptance.

## Discussion

Herein, we examined the neural correlates of perceived social support which was determined with the SPS (Cutrona and Russell, 1987), and further explored its relationship with psychological well-being. The whole brain analysis revealed that perceived social support was correlated with the left MTG GM volume. What’s more, the left MTG GM volume observed above was also correlated with psychological well-being, and perceived social support mediated the link between the left MTG GM volume and psychological well-being.

First, our study revealed that perceived social support was positively correlated with the left MTG GM volume, that is, the larger left MTG GM volume, the greater perceived social support. MTG was a key brain region involved in semantic processing, language comprehension, face emotion recognition, empathy, and theory of mind (Meyer et al., 2005; Vollm et al., 2006; Lindenberg and Scheef, 2007; Acheson and Hagoort, 2013; Lai et al., 2016; Yun et al., 2017; Yuk et al., 2018). These social cognitive processes play important roles in daily social interactions, in which people understanding other’s language, recognize other’s facial expressions, and infer other’s psychological states and intentions (Frith, 2009). Therefore, it is reasonable that MTG is closely related to people’s social interactions, and previous studies have shown that social network size was correlated with the structure (GM volume/GM density/ volume) of MTG (Kanai et al., 2011; Blumen and Verghese, 2019; Taebi et al., 2020). Further, good social interactions may contribute to more perceived social support through multiple ways. Specifically, social interactions may provide individuals with more opportunities to express their thoughts, feelings, and emotions, leading to receive more emotional support (Kafetsios and Nezlek, 2012); they may increase opportunities for cooperation, so that individuals may have access to more resources for assistance (Duffy and Ochs, 2009); and they may increase an individual’s sense of belonging to the group, which may lead to greater confidence in the availability of help from others in the group (Guo and Cheng, 2016; Ahn and Davis, 2020). Therefore, individuals with a larger left MTG GM volume may perceive more social support. In fact, a recent study using older adults has found that the more perceived social support measured by the MOS-SSS, the greater GM volume of MTG (Cotton et al., 2020). Our study using the SPS to measure perceived social support in young adults found similar results, which extended previous studies and further confirmed the role of MTG for social interaction and social support.

Besides, this study also showed that individuals with more perceived social support had better psychological well-being, which was consistent with numerous previous behavioral studies (Huppert, 2010; Orehek and Lakey, 2011; Lakey, 2013; Kalpana, 2016; Szkody and Mckinney, 2019). It was worth mentioning that perceived social support showed a high correlation with psychological well-being in our study. This may be for the reason that Chinese participants were used in this study. In Eastern cultures, where collectivist values are emphasized, persons value harmony and equality among people, and having good interpersonal relationships is an important source of individual well-being (Lu et al., 2001; Joshanloo, 2013); whereas in Western individualistic cultures, where personal values are more important, the achievement of personal goals is an important influence factor for individual well-being (Lu et al., 2001; Joshanloo, 2013). In fact, studies have shown that Westerners’ happiness is closely related to independence, while Easterners’ happiness is related to interdependence (Gardiner et al., 2020).

More importantly, the present study also found that the left MTG GM volume was associated with psychological well-being, and perceived social support mediated the link between the left MTG GM volume and psychological well-being. Although previous studies have demonstrated that MTG was related to psychological well-being (Lewis et al., 2013; Luo et al., 2017; King, 2019), no study has revealed why MTG is related to psychological well-being. The present study provided a possible explanation that individuals with larger MTG GM volumes perceived more social support, while good social support played a significant role in enhancing individuals’ psychological well-being.

Of course, the study also has limitations. Firstly, the present study only revealed the relationship between GM volume of the brain and behavioral variables, and the future study can combine multimodal imaging techniques to reveal the neural correlates of behavioral variables more comprehensively. Secondly, the current study was only a correlational study and could not reveal the causal relationship between variables, which needs to be addressed with longitudinal studies in the future. Third, the current study only included sex, age, SES and the total GM volume as covariates when building the statistical model, and future studies need to include more covariates (e.g., race, number of friends, self-esteem, psychological resilience, etc.) in order to obtain more stable results. Fourth, the participants in this study were all young college students, which limited the generalization of the present findings, and further validations of the findings in other populations are needed in the future. Fifth, the present study only measured perceived social support, and future studies could explore the differences in the neural correlates of perceived social support and actual social support. Finally, future studies could further explore the relationship between perceived social support and different types of well-being (e.g., subjective well-being, social well-being).

## Data availability statement

The raw data supporting the conclusions of this article will be made available by the authors, without undue reservation.

## Ethics statement

The studies involving humans were approved by Institutional Review Board of Beijing Normal University. The studies were conducted in accordance with the local legislation and institutional requirements. The participants provided their written informed consent to participate in this study.

## Author contributions

HL: Conceptualization, Formal analysis, Funding acquisition, Methodology, Validation, Visualization, Writing – original draft, Data curation, Investigation, Project administration, Resources, Software, Writing – review & editing. YS: Conceptualization, Funding acquisition, Project administration, Resources, Validation, Visualization, Writing – original draft, Formal analysis, Methodology, Supervision, Writing – review & editing. XW: Data curation, Formal analysis, Investigation, Methodology, Software, Writing – review & editing. JL: Project administration, Resources, Supervision, Writing – review & editing.
